# Increasing serum antibodies against type B influenza virus in 2017–2018 winter in Beijing, China

**DOI:** 10.1186/s13568-022-01469-9

**Published:** 2022-10-01

**Authors:** Yao Yao, Lingling Chen, Dong Zhu, Runqing Li, Zhipeng Zhao, Wenqi Song, Xiuying Zhao, Kun Qin

**Affiliations:** 1 Department of Clinical Laboratory, Beijing Children’s Hospital, Capital Medical University, National Center for Children’s Health, Beijing, 100045 People’s Republic of China; 2 National Institute for Viral Disease Control and Prevention, China CDC, Key Laboratory for Medical Virology, National Health Commission, 100 Yingxin Street, Beijing, 100052 People’s Republic of China; 3 Department of Clinical Laboratory, Beijing Tsinghua Changgung Hospital, School of Clinical Medicine, Tsinghua University, Beijing, 102218 People’s Republic of China; 4 Wuhan Center for Disease Control and Prevention, Wuhan, 430024 Hubei People’s Republic of China

**Keywords:** Type B influenza virus, Hemagglutinin inhibition (HI) antibodies, Geometric mean titer (GMT)

## Abstract

Influenza B virus circulates yearly with lower activity than that of influenza A virus in China. During winter 2017 to 2018, a sharp surge of influenza activity dominated by type B/Yamagata lineage virus caused unprecedented medical burden in Beijing. This research aimed to understand the underlying mechanism for this circulation and prepare for epidemics in the future. Sera samples collected from the patients in 2016–2017 and 2017–2018 flu seasons were tested for profiling hemagglutinin inhibition (HI) antibodies against both prevailing Victoria and Yamagata lineages of type B influenza viruses. It showed that the seroprevalence against both lineages of the virus in 2017–2018 winter was higher than that in 2016–2017, while no difference of the seroprevalence was observed between the two viruses. Meanwhile, significant elevated geometric mean titer (GMT) against both lineages of influenza B viruses was found in the specimens collected during 2017–2018 flu season than that from 2016 to 2017, suggesting the viruses might undergo antigenic changes. These results also suggested that lower GMT against both type B variants in 2016–2017 might serve as an immunological niche for the dominating of B/Yamagata virus in China during 2017–2018 winter season. Our findings have implication that there was a significantly elevation of HI antibodies to influenza viruses B in 2017–2018 than in 2016–2017. On the other hand, the low level of HI antibodies to both B/Y and B/V in 2016–2017 could contribute to the severe B/Y epidemic in 2017–2018 to some extent.

## Introduction

Since October 2017, A high incidence of influenza activity was reported by the World Health Organization (WHO) (WHO 2018). Compared with previous flu seasons, it surged at a high level till April, 2018 and caused a huge medical burden in Beijing (Kochanek et al. 2018). Sharply increasing number of patients with influenza-like-illness (ILI) suggested that the circulating virus might have evaded the herd immunity or mutated to achieve a higher transmissibility (Bedford et al. 2015). This predication soon was confirmed by the evidence from China CDC that Yamagata lineage of type B influenza virus (Flu B) was the predominant strain resulting the epidemic, although few cases were reported to be type A influenza virus (Flu A) infection including pdmH1N1/2009 and H3N2 (CDC 2018).

Flu B virus was firstly identified in 1940 and was associated with considerable hospital admissions and deaths worldwide annually (FRANCIS 1940; Chen and Holmes 2008). During early 1980s, it evolved into two lineages, designated as B/Yamagata (B/Y) and B/Victoria (B/V), showing distinct antigenicity and transmission pattern (Chen and Holmes, 2008; Kanegae et al. 1990; Vijaykrishna et al. 2015). Since then, Flu B viruses have co-circulated with Flu A virus during epidemic season and the dominant lineage has changed over years in different geographical locations (Ye et al. 2018; Yang et al. 2018). Several studies have pointed out the differences of the epidemiology between B/Y and B/V lineages, such as younger average ages of persons could be more easily attacked by higher transmissibility of B/V viruses, compared with B/Y viruses (Vijaykrishna et al. 2015; Caini et al. 2019; Yoshihara et al. 2019).

For a long time, many researches have been focused on Flu A virus, whereas Flu B virus was relatively less investigated, resulting in poor lineage matches with recommended influenza vaccine strains, and the control options require further improvement (Htwe et al. 2019; Noh et al. 2018). HI tests measure the presence of specific antibodies of IgG in sera that inhibit virus-mediated agglutination of erythrocytes (Rowe et al. 1999). It is a sensitive assay that is affected, in some level, by nonspecific hemagglutinin inhibitors in the sera. However, it is a particularly reliable method for influenza surveillance, providing confirmative result for susceptive individuals with successive sera samples, also profiling the herd immunity against upcoming influenza variant, in which case titers ≥ 1:40 are considered protective (Skevaki et al. 2012). In the present study, serological assay against two lineages of Flu B viruses circulating in China was determined to investigate the cross-sectional HI antibodies in defined samples. Our findings provided insights that low level herd immunity to influenza B virus prior to the B/Y lineages epidemic would in some extent contribute to this epidemic.

## Materials and methods

### Sera samples and ethics approval

Two batches of serum samples from Beijing Tsinghua Changgung Hospital (BTCH) and Beijing Children Hospital (BCH), respectively, were used in the present study. Batch one was 190 sera samples collected during Nov, 2016 to Apr, 2017 (2016–2017) from outpatients in BTCH with the age ranging from 0.5 to 94 years old. Batch two was 283 sera samples collected from outpatients presenting acute respiratory infections (ARI) from BCH during the Dec, 2017 to Mar, 2018 (2017–2018), with the age ranging from 0.5 to 17 years old. Samples in Batch one was subdivided into 3 groups according to ages and diagnosis, group 1 from 69 adults aged from 18 to 94 years old and excluded respiratory diseases, group 2 from 75 patients aged from 0.5 to 17 years old present other than respiratory symptoms, group 3 from 46 patients aged from 0.5 to 17 years old and presenting ARI. All sera samples were remaining from routine clinical tests and were stored at − 40 °C till use.

### Representative influenza viruses for HI assay

**Fig. 1:**
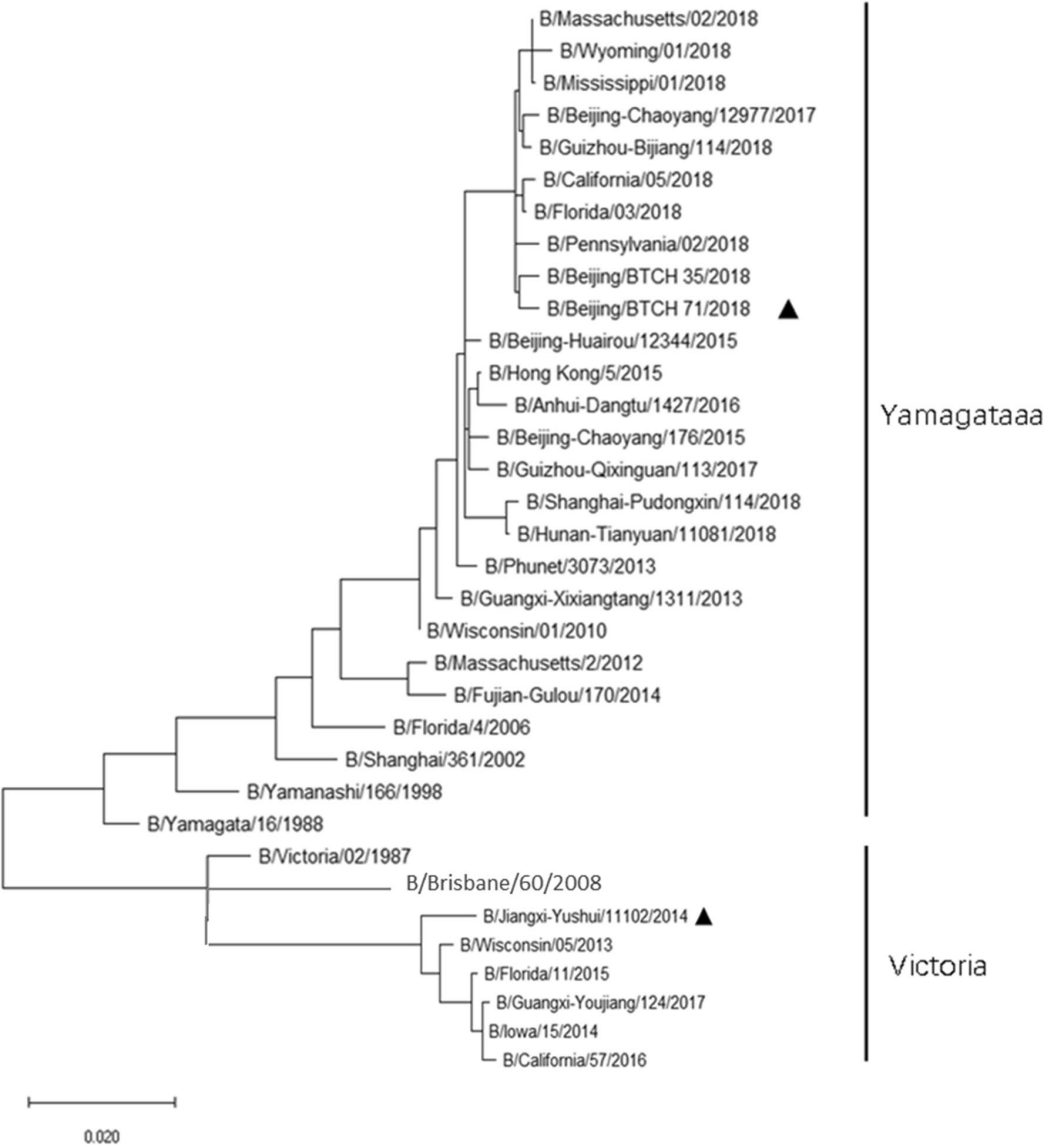
Phylogenetic tree of the HA gene of type B influenza viruses used in the study. Phylogenic tree was constructed using the partial HA genes (370-610NT covering the most important positions distinguishing the Victoria and Yamagata lineages) from the clinic isolates and full-length HA sequences from the GenBank as well as GISAID. Strains labeled with black triangle represented as B/Victoria and B/Yamagata lineage viruses were used as antigens in the present study

Type B influenza viruses isolated from China during the two winter seasons were selected for HI assay on the basis of data collected by the World Health Organization (WHO) as well as our own study (Zhu et al. 2019). For Victoria lineage, B/Jiangxi_yushui/11102/2014 which was phylogenetically and antigenically close to the recommended vaccine strain B/Brisbane/60/2008, belonging to V1A sublineage, was selected as the antigen for HI. For Yamagata lineages, the representative virus B/Beijing/BTCH_71/2018, belonging to Y3 sublineage isolated in early 2018 in Beijing, was used (Zhu et al. 2019). The phylogenetic relationship of the selected viruses was presented (Fig. 1). Viruses were amplified in 9–11-day-old specific pathogen free (SPF) chicken embryonated eggs at 35 °C for 2 days. The allantoic fluid were collected and cleared by low-speed centrifugation. The titer of the virus was determined by HA assay, and viral antigen aliquots were stored at − 80 °C till use. All experiments associated with live viruses were performed in the biosafety level 2 plus laboratory.

### Hemagglutinin inhibition (HI) assay

Serum samples were examined for the presence of antibodies against the hemagglutinin of the selected type B influenza viruses by HI assay as described previously (Bodewes et al. 2011; Wang et al. 2012). Briefly, serum samples were heat inactivated at 56 °C for 1 h and then were absorbed by 20% (v/w) turkey red blood cell (TRBC) to reduce the non-specific binding. twofold serial dilutions with 1:10 staring dilution of pretreated serum samples were subsequently incubated with 8 hemagglutination units of influenza virus or phosphate-buffered saline (PBS) for 30 min at 37 °C, and subsequently, 1% TRBC was added. HI pattern was read after incubation for 30 min at 22 °C. The highest dilution of serum that still gave complete inhibition of the hemagglutination was recorded as the titer. Serum samples were considered negative when they completely failed to inhibit agglutination of TRBC by any of the selected viruses. Serum samples collected from mice before and after immunization with each of the type B influenza viruses were used as negative and positive controls, respectively. Considering previous exposures to Flu B viruses or vaccinations in individual’s life would have interference on HI antibody profile, we took ≥ 1:80 as cut-off limit for “positive reaction” of recently infection.

### Statistics

Data analyses were performed using SPSS software (version 17.0; SPSS Inc., Chicago, IL, USA). Nonparametric test was used for comparing the age difference of children and GMT of the sera samples. Correlation of HI titers against two lineages of Flu B virus was employed Spearman tests. The Pearson Chi-square test or Fisher’s exact tests were performed to evaluate HI antibodies seroprevalence to Yamagata and Victoria lineages of Flu B virus, with cut-off value as HI titers ≥ 1:80. Two-sided at the 5% level of significance was considered statistically remarkable in all tests.

## Results

### HI titers against two lineages of Flu B viruses in sera from 2016–2017

The HI titers to the two lineages of Flu B viruses in sera from 2016–2017 were compared. Result indicated that the frequency with HI titers over the cut-off value in the 3 subgroups did not show difference, either to B/Y, or to B/V lineage virus (Table 1). HI titers in group 3, the sera from children present ARI did not increase as compared to the group 2, sera from children without ARI (*P* = 0.47 for B/Y and *P* = 0.78 for B/V). Hence, we put group 2 and group 3 together (n = 121) to represent HI value in children (CHI) from 2016–2017. The HI titers in CHI group were also compared to subgroup 1 of adults, and no difference was found (*P* = 0.65 for B/Y and *P* = 0.49 for B/V). The results indicated a lower epidemic of Flu B in 2016–2017 winter, with the HI titers in children and adult showing no difference previous the B/Y epidemic year.

**Table 1:** Summary and comparing of HI titers to B/Y and B/V lineages in 2016–2017

Sera cohorts	Group1 (N = 69) No. (%)	Group2 (N = 75) No. (%)	Group3 (N = 46) No. (%)	*P* value (3 groups)	*P* value (group 2 to 3)	*P* value (CHI to adults^†^)
B/Yamagata^a^	14 (20.29)	19 (25.33)	9 (19.57)	0.69	0.47	0.65
B/Victoria^b^	13 (18.84)	18 (24.00)	10 (21.74)	0.76	0.78	0.49
*P* value	1.00	1.00	1.00	–	–	–

The percentages were estimated as a fraction of total cases belonging to each category. P values were estimated using χ^2^ tests or Fisher’s exact tests (between B/Y and B/V of each group)

^†^CHI: summary of group 2 and group 3 to represent samples from children. Adults: samples from adult in group 1

^a^B/Beijing/BTCH_71/2018 was used as antigen representative of B/Yamagata lineage virus

^b^B/Jiangxi_Yushui/11102/2014 was used as antigen representative of B/Victoria lineage virus

### Comparing HI titers against two lineage of Flu B viruses in 2016–2017 and 2017–2018

**Fig. 2:**
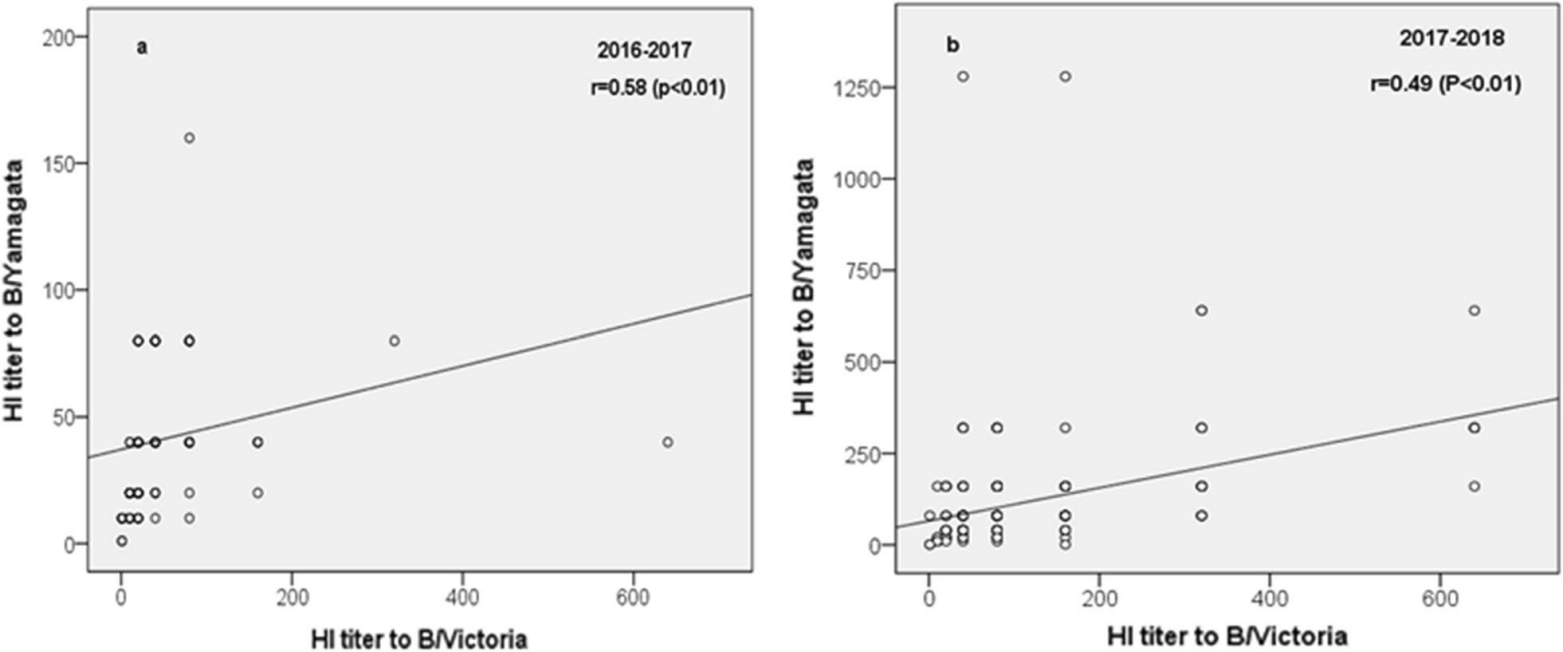
Comparison of HI titer distributions against two lineages of Flu B viruses by Spearman tests. We can see categories of HI titer to both lineages increased significantly in 2017–2018 than in 2016–2017. Moderate correlation can be found between B/Y and B/V lineage as the HI titers in 2016–2017 (**a**) and 2017–2018 (**b**)

HI titer against prevailing Flu B viruses was compared using sera samples from 2016–2017 and 2017–2018. All the samples come from children aged from 0.5 to 17 years, and no difference were found (*P* = 0.77). There were 28 sera samples in 2016–2017 CHI (n = 121) groups being found as HI positive against both lineages of Flu B viruses, with the frequency at 23.14%, and no difference was found between B/Y and B/V lineage. However, there were 66.08% samples showed HI positive against B/Y lineage and 59.72% samples showed HI positive against B/V lineage in the 2017–2018, with no significant difference were observed to B/Y and B/V lineages in this season, though the HI positivity to B/Y lineage did mildly increase than B/V (66.08% vs 59.72%, *P* = 0.054). It was significantly increased as the HI titers in 2017–2018 than in 2016–2017, either to B/Yamagata (*P* < 0.01) and B/Victoria (*P* < 0.01), indicating that HI antibody in children increased simultaneously, to both B/Y and B/V viruses with the spreading of B/Y lineage (Table 2). On the other hand, a moderate correlation was found between HI titers to B/V and B/Y in 2016–2017 (*r* = 0.58) and 2017–2018 (*r* = 0.49) (Fig. 2).

**Table 2:** Comparing prevalence of HI titers to B/V and B/Y lineages in 2016–2017 and 2017–2018

Sera cohort/antigen	2016–2017(N = 121) No. (%)	2017–2018(N = 283) No. (%)	*p* value
B/Yamagata^a^	28 (23.14%)	187 (66.08%)	< 0.01
B/Victoria^b^	28 (23.14%)	169 (59.72%)	< 0.01
*P* value	1.00	0.054	–

The percentages were estimated as a fraction of total cases belonging to each category. P values were estimated using χ^2^ tests or Fisher’s exact tests (age groups), and p values of 0.05 or less are shown in bold

^a^B/Beijing/BTCH_71/2018 was used as the representative of B/Yamagata lineage virus

^b^B/Jiangxi_Yushui/11102/2014 was used as the representative of B/Victoria lineage virus

### Antigenic drift of Flu B viruses from 2016–2017 to the 2017–2018

Geometric mean titer (GMT) was usually used for evaluating the influenza vaccine efficacy and potency in defined population. In this study, the GMT of HI titer against both lineages of Flu B viruses in sera collected in 2017–2018 (74.16 against B/Y and 67.57 against B/V, respectively) was much higher than that collected in 2016–2017 (31.87 against B/Y and 26.44 against B/V, respectively). Significant increase in the GMT to both lineages could be seen in 2017–2018, as compared to 2016–2017. However, we did not find difference as the GMT to B/Y and B/V either in 2016–2017 (*P* = 0.14), or in 2017–2018 (*P* = 0.11). Given the same antigens used in the HI assay for sera samples from two different years, we predict that Flu B viruses might undergo antigenic drift and it is the reason for the B/Y lineage virus evades herd immunity. Collectively, these data suggested weak HI response in 2016–2017 to both Flu B viruses may contribute to the severe epidemic of Flu B in winter of 2017 (Table 3), and the antigenic drift produce a transition and higher HI titers against most recent/homologues infection.

**Table 3:** GMT of HI titers against two lineages of Flu B viruses in sera from children in 2016–2017 and 2017–2018

Sera cohorts	B/Yamagata^a^ (95% CI^c^)	B/Victoria^b^ (95% CI^c^)	*P* value
2016–2017 (N = 121)	31.87 (27.56–36.86)	26.44 (21.54–32.46)	0.14
2017–2018 (N = 283)	74.16 (66.39–82.83)	67.57 (60.51–75.44)	0.11
*P* value	** < 0.01**	** < 0.01**	

GMT was calculated based on the HI titer. P values were estimated using nonparametric tests and p values of 0.05 or less are shown in bold

^a^B/Beijing/BTCH_71/2018 used as the representative of B/Yamagata lineage virus

^b^B/Jiangxi_Yushui/11102/2014 used as the representative of B/Victoria lineage virus

^c^*CI* confidence interval

## Discussion

HI assay is accepted as a standard laboratory procedure for the classification or subtyping of hemagglutinating viruses. For influenza virus, HI assay is adapted to identify the hemagglutinin subtype specificity of antibodies to influenza virus (Pedersen et al. 2014). In the context that there were an abrupt spread of influenza B/Y lineage virus in 2017–2018 winter, we performed HI antibody screening using cryopreserved sera samples, aimed to evaluate the HI antibody transition after the outbreak of B/Y lineage. By setting 1:80 as cut-off value, the positivity of HI titer and GMT in both B/Y and B/V lineages were compared between the year of 2017–2018 to previous year of 2016–2017. Results indicated that the positivity of total HI titer in 2017–2018 was significantly higher than that in 2016–2017, and the GMT was also significantly increased in 2017–2018 than in the previous year. However, we did not find any difference in HI titer between B/Y and B/V in the two consecutive years. In 2016–2017, positivity and GMT of HI titer for both B/Y and B/V were at a low level. Conversely, in 2017–2018, the two indicators increased simultaneously. It was indicated in many reports that B/Y prevalence account for severe spreading of influenza during that time (Dayan. 2018). The HI antibody to B/Y lineage with percentage of 66.1%, far exceeds reported positivity of antigen tests to Flu B at around 10–20%, with research proved it that positivity of RT-PCR assay to Flu B at 38% in ILI cases during 2017–2018 winter (Zhu et al. 2019), suggesting recessive infection would be a primary reason for acquiring the HI antibodies under Flu B epidemic.

Throughout the years, influenza A viruses have attracted a great deal of attention and have caused several global pandemics due to their strong transmission capacity and great variability. In contrast, there is less literature focused on the epidemiology and societal burdens of influenza B (Caini et al. 2015). Sudden outbreak of Flu B/Y virus during the 2017–2018 winter may reflect changes of the viral fitness (Virk et al. 2019), as well as low herd immunity against this leading strain. The B/Y variant become to dominate might reflect the vaccine effectiveness (VE) or natural situation cannot provide adequate protective immunity against this strain. In this study, low GMT of HI titer level of B/Y and B/V in previous epidemic year (2016–2017) add value to this assumption and provide a platform to the emergence of the B/Y epidemic. HI titer could not only serve as an indicator for the protective immunity after immunization, but also hint infection/transmission status in a well-defined population (Bodewes et al. 2011). Flu B HI titer was detected at very low incidence in ILI patients during 2018–2019 seasonal flu epidemic (unpublished data by our group), which could be attributed to previous year’s high incidence of HI antibodies to B/Y and B/V lineages.

In China, the public has gradually developed a strong “vaccine hesitancy”. Individuals even those with high risk are reluctant or refuse to be vaccinated despite the availability of influenza vaccine. Influenza vaccination coverage rate in China was at 1.5% of the total population between 2004 and 2014 (Yang et al. 2016). It can be presumed the establishment of herd immunity against influenza virus mainly relies on the passive immunization by natural infection. Low immunization coverage and lack of B/Y antigen in current trivalent influenza vaccine (TIV) in China (before 2018–2019 flu season) may offer access to the dominance and prevalence of B/Y virus. Long-term co-circulating with viruses of Flu A H1/H3 subtypes, accompanied by decreased HI antibody to both Flu B virus strains may serve as an immunological niche for the B/Y lineage virus spreading in China during the 2017–2018 winter. Our findings have implications that quadrivalent influenza vaccines (QIV) should be timely updated for better prevention against seasonal influenza epidemics.

It has been well documented that antisera raised against Victoria lineage of Flu B virus shared low cross reaction with that against Yamagata lineage (Rota et al. 1992). However, whether the distinct immunodominance of the two lineages of Flu B virus could affect or elicit more potent cross react or protective antibodies based on previous research of infection needs in-depth investigations. Same situation had been occurred with Flu A as previous seasonal H1N1 strain was replaced by pdm/H1N1/2009 virus, and inducing cross protection antibody response in infections (Victora and Wilson, 2015; Andrews et al. 2015). On the background, HI assay could be used as evaluation of infection and herd immunity but not suitable for diagnosis in clinical for cross reaction between B/Y and B/V lineages from previous infection could not be excluded.

In summary, our study indicates that there was a significantly elevation of HI antibodies to influenza viruses B in children with ARI in 2017–2018, compared with children in 2016–2017. On the other hand, the low level of HI antibodies to both B/Y and B/V in 2016–2017 could contribute to the severe B/Y epidemic in 2017–2018 to some extent. We aimed to offer an insight into the dynamics of FluB viruses epidemiology. Serology results provide important context for B/Yamagata virus may act as the predominate strain attacking children during 2017–2018 flu season. This results also were convinced from phylogenetic analyses of HA genes (Yan et al. 2020). Limitations exist in the study, First of all, we used preserved clinical samples from children with ARI to perform the cross-sectional study, the sera were not so typical and previous infection or vaccination might affect production of HI titers. Furthermore, the sera samples preserved in 2016–2017 were not sufficient enough, we had to make a composition using samples from children with or without ARI together, after excluding difference between the two groups, and to compare to those from 2017–2018. At last, since we did not perform HI assay to Flu A and other strain of Flu B/Y, we are not sure the influence of HI reaction to Flu B.

## Data Availability

All relevant data are within the manuscript.
